# Oxidative Stress and Inflammatory Biomarkers in Patients with Diabetic Foot

**DOI:** 10.3390/medicina58121866

**Published:** 2022-12-17

**Authors:** Sanja Vujčić, Jelena Kotur-Stevuljević, Jelena Vekić, Iva Perović-Blagojević, Tatjana Stefanović, Sanja Ilić-Mijailović, Branka Koprivica Uzelac, Srećko Bosić, Tamara Antonić, Azra Guzonjić, Ana-Marija Mastilović, Zorica Marković, Manfredi Rizzo

**Affiliations:** 1 Department of Medical Biochemistry, University of Belgrade-Faculty of Pharmacy, 11000 Belgrade, Serbia; 2 Clinical Hospital Center “Dr Dragiša Mišovic—Dedinje”, 11000 Belgrade, Serbia; 3 General Hospital Požarevac, 12000 Požarevac, Serbia; 4 Department of Health Promotion, Mother and Child Care, Internal Medicine and Medical Specialties, University of Palermo, 90100 Palermo, Italy

**Keywords:** diabetic foot, oxidative stress, diabetes mellitus, PAB, NLR

## Abstract

*Background and Objectives*: Diabetic foot (DF) development is driven by complex interactions of hyperglycemia, inflammation, and oxidative stress (OS). We aimed to investigate OS and inflammatory biomarkers in patients with DF and their potential to improve early diagnosis and management of DF. *Materials and Methods*: The prooxidant–antioxidant balance (PAB), superoxide dismutase (SOD), total oxidative status (TOS), total sulfhydryl groups (SHG), routine biochemical parameters, and complete blood count were determined in 42 patients with type-2 DM, of which 23 patients had DF, while 19 patients were without DF complications. The neutrophils-to-lymphocyte ratio (NLR) was evaluated as a biomarker of inflammation. *Results*: Patients with DF had significantly higher (*p* < 0.05) PAB levels (170 ± 33.9 U/L) compared to those without DF complications (142 ± 31.3 U/L). In addition, patients with DF had significantly reduced SOD activities (*p* < 0.01). NLR values were significantly higher in the DF group (median: 2.8; interquartile range: 2.0–4.3) than in the group without DF (median: 1.4; interquartile range: 1.4–2.1; *p* < 0.01). A positive correlation was found between the PAB and NLR index (r = 0.449; *p* < 0.05). The diagnostic accuracy of both PAB (AUC = 0.741; *p* < 0.01) and NLR (AUC = 0.760; *p* < 0.01) was estimated as acceptable. *Conclusions*: In conclusion, the development of DF is associated with enhanced OS and inflammation processes. PAB and NLR could be useful non-invasive biomarkers of DF development.

## 1. Introduction

Type-2 diabetes mellitus (T2DM) is a leading cardiometabolic disorder of the modern age. Despite constant advances in preventive medicine, the global incidence of T2DM remains high [1]. Furthermore, T2DM is often diagnosed too late, when one or more complications have already developed [2,3]. Diabetic foot (DF) is one of the most severe complications of diabetes [4], which considerably worsens the quality of life of the patients [2]. Without adequate early intervention, ischemic foot changes may progress to necrosis and gangrene of the foot [2]. Hence, DF is considered to be the major cause of non-traumatic lower limb amputations [5]. In addition, patients with DF are characterized by increased morbidity and mortality due to cardiovascular disease (CVD) [6]. These data imply that the development of DF and atherosclerosis share common mechanisms. 

Evidence-based guidelines consider long-standing diabetes, poor metabolic control, smoking, and the presence of other microvascular and macrovascular complications as the main risk factors for DF development [1,2]. In addition, patients with diabetes are also characterized by enhanced oxidative stress (OS) [7,8,9], which plays an active role in the pathogenesis of complications [10]. Recent studies suggest that patients with DF have increased levels of serum prooxidants, including malondialdehyde (MDA) and prooxidative–antioxidative balance (PAB) [11,12,13]. Furthermore, a depletion of antioxidative mechanisms has been demonstrated in both wound tissue [14] and blood samples [15] of patients with DF. Although serum OS biomarkers are widely used as a non-invasive approach for a redox status assessment, their clinical importance in the etiology of DF has been less studied. To overcome the gaps in the literature, further studies are necessary.

The hyperglycemia-induced overproduction of reactive oxygen species (ROS) significantly contributes to endothelial dysfunction and inflammation [10]. Earlier studies have demonstrated increased levels of inflammatory biomarkers, such as high sensitivity C-reactive protein (hsCRP), procalcitonin, and interleukin-6, in serum of patients with DF [16,17]. As recently suggested, these biomarkers could add diagnostic and prognostic information in terms of concomitant infection and the risk of amputation and post-operative mortality [18]. The neutrophil-to-lymphocyte ratio (NLR) is a readily available and economical biomarker, and a growing body of evidence suggests its clinical significance in patients with cardiometabolic diseases [19]. In line with the previous information, Duman et al. [20] demonstrated increased NLR in patients with poorly regulated T2DM. Recently, a higher NLR index has been demonstrated in patients with DF [21]; moreover, a positive correlation has been reported between elevated NLR and osteomyelitis, increased risk of amputation, and septic complications in patients with DF ulcerations [22]. 

The aim of this study was to examine the significance of OS and inflammatory biomarkers determination in patients with DF, as well as to investigate their potential to improve early diagnosis and management of DF.

## 2. Materials and Methods

### 2.1. Study Participants

This cross-sectional study included 23 patients who were consecutively diagnosed as having DF based on a medical history and standard foot examination according to the national guidelines [2]. These patients were hospitalized at the surgical department of the General Hospital in Požarevac between December 2020 and March 2021 in order to assess the requirement for amputation. Thirteen patients underwent surgery, while the remaining ten were treated conservatively. The study also included 19 patients with T2DM who were treated at the Clinical Hospital Center “Dr Dragiša Mišović” in Belgrade between April 2019 and March 2020. Additional inclusion criteria for patients treated at the Clinical Hospital Center “Dr Dragiša Mišović” was poor metabolic control and no previous history or clinical evidence of DF complications. The exclusion criteria for both groups of patients were previous history of COVID-19, which has been confirmed to affect inflammation, OS, and the further course of the underlying disease. 

The following demographic and clinical data were collected: age, gender, smoking status, duration of diabetes, presence of comorbidities (hypertension, CVD), and prescribed medications, including oral antidiabetic agents (OAD) and insulin. Following the international guidelines, hypertension was defined as systolic blood pressure (SP) ≥ 130 mmHg and/or diastolic blood pressure (DP) ≥ 80 mmHg and/or current antihypertensive therapy, and obesity was considered in patients who had a body mass index (BMI) ≥ 30 kg/m^2^, while the presence of anemia was determined if hemoglobin (Hgb) levels were <135 g/L in men or <120 g/L in women [23,24]. The study was conducted under the ethical principles of the Declaration of Helsinki with the approval of the Ethics Committees of the Clinical Hospital Center “Dr Dragiša Mišović” and the General Hospital in Požarevac. All patients gave written, informed consent.

### 2.2. Laboratory Analyses

Blood samples were obtained after the overnight fasting period. In DF patients who required amputation, the sampling was performed before the surgery. The serum glucose concentration was measured by a standard enzymatic method on the Dimension RxL Max analyzer (Siemens Health Care Diagnostics, Erlangen, Germany). The level of glycohemoglobin (HbA_1c_) was determined by the immunoturbitimetric method on the Innova Star (DiaSys Diagnostic Systems, GmbH, Germany) and Dimesion EXL (Siemens Health Care Diagnostics, Erlangen, Germany) analysers. Complete blood count (CBC) was obtained using the hematologic analyser XN-1000 (Sysmex Corporation, Kobe, Japan). The NLR index was calculated as a ratio of absolute neutrophil-to-leukocyte counts. Redox status assessment included determination of total oxidative status (TOS), prooxidative–antioxidant balance (PAB), SOD activity, and total sulfhydryl groups (SHG) content by spectrophotometric methods, as previously described [25,26,27,28].

### 2.3. Statistical Analysis

The Shapiro–Wilk test was used for testing normality of the data. Normally distributed continuous variables were presented as arithmetic means with standard deviations, while skewed data were presented as medians and interquartile ranges. Normally distributed continuous variables were compared with the Student’s *t* test, while skewed variables were compared using the Mann–Whitney U test. Chi-square and Fisher Exact tests were used to analyze categorical variables. Spearman’s non-parametric correlation analysis was used to investigate associations between OS and inflammatory and metabolic control biomarkers. Binary logistic regression analysis was used for testing the association of PAB and NLR with DF, while ROC (Receiver Operating Characteristics Curve) analysis was performed to test diagnostic accuracy of these parameters. The discriminatory ability of the test was evaluated according to the Hosmer and Lemeshow criteria. Based on the area under the ROC curve (AUC), the models can be classified as: weak (0.5 ≤ AUC < 0.7), acceptable (0.7 ≤ AUC < 0.8), excellent (0.8 ≤ AUC < 0.9), or extraordinary (AUC ≥ 0.9). The probability *p* ≤ 0.05 was taken as a condition for the existence of statistical significance. SPSS software (version 18.0 for Windows, SPSS Inc., Chicago, IL, USA) was used for statistical analysis.

## 3. Results

The demographic and clinical data of patients are shown in Table 1. Patients with DF were older than patients without DF (*p* < 0.05), while the distributions of gender, smokers, and patients with hypertension and obesity were similar. The group with DF was also characterized by a longer duration of diabetes (*p* < 0.05) and a higher prevalence of patients treated with insulin (*p* < 0.001). An evaluation of biochemical parameters showed that glucose concentration did not differ significantly between the groups, while the level of HbA_1c_ was significantly lower in patients with DF (*p* < 0.05). 

We further analyzed the complete blood count (CBC) parameters in both groups. As shown in Table 2, hemoglobin (Hgb) levels and red blood cell (RBC) counts were significantly lower (*p* < 0.05) in patients with DF. Accordingly, a significantly higher prevalence of patients with anemia was presented in the DF group (60.9% in the DF group vs. 20.3% in the group without DF; *p* < 0.001). An analysis of the distribution of white blood cell (WBC) subpopulations showed significantly increased neutrophil and reduced lymphocyte counts in patients with DF. In line with the previous findings, the NLR ratio was significantly higher in the DF group (*p* < 0.01).

**Figure 1:**
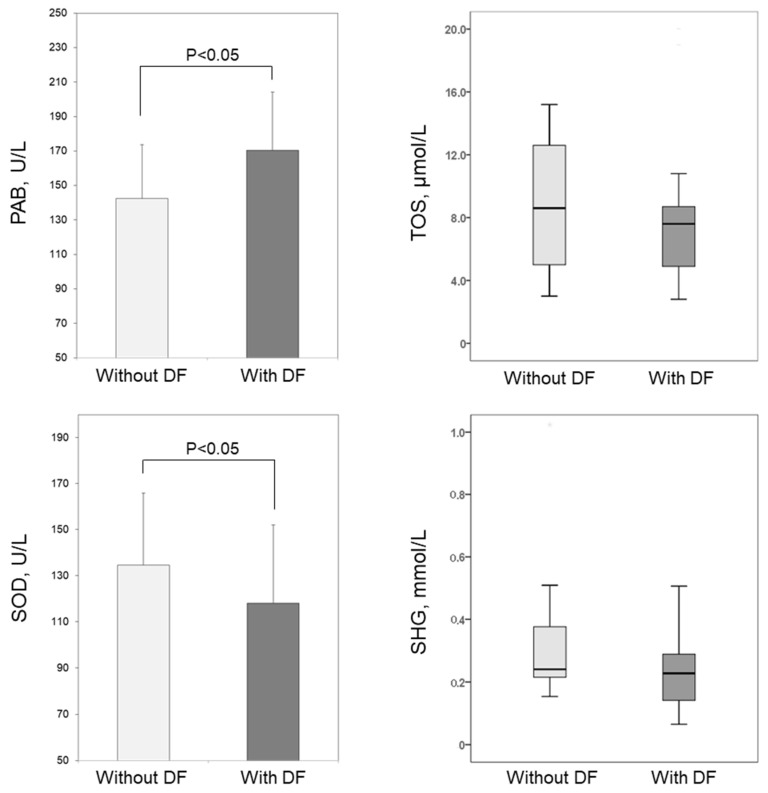
Biomarkers of OS in patients without DF and with DF. The levels of PAB and SOD were presented as means and standard deviations, and the levels of TOS and SHG as medians and interquartile ranges. Abbreviations: PAB, prooxidative-antioxidative balance; SOD, superoxide dismutase; TOS, total oxidative status; SHG, total sulfhydryl groups.

The parameters of the redox status were presented in Figure 1. We found that the levels of PAB in the DF group (170.4 ± 33.9 U/L) were significantly higher than the levels (142.5 ± 31.2 U/L) in patients without DF (*p* < 0.01). On the other hand, superoxide dismutase (SOD) activities were significantly lower (*p* < 0.05) in patients with DF (118.1 ± 13.6 U/L) as compared to patients without DF complications (134.6 ± 13.7 U/L). The levels of total oxidative status (TOS) and total sulfhydryl groups (SHG) did not differ significantly between the groups (*p* = 0.397 and *p* = 0.123, respectively).

Further analysis of the DF group showed that levels of PAB (182.4 ± 26.7 U/L) in patients who underwent amputation (N = 13) were higher than the levels (154.9 ± 37.2 U/L; *p* = 0.05) in patients who did not require amputation (N = 10). In addition, DF patients who underwent amputation had significantly reduced levels of SHG (median: 0.15 mmol/L; interquartile range: 0.12–0.28 mmol/L; *p* < 0.01) as compared to those who did not undergo the surgery (median: 0.29 mmol/L; interquartile range: 0.26–0.35 mmol/L). No difference was found in NLR values between the groups (*p* = 0.203).

Next, we examined the association of OS biomarkers with parameters of inflammation in patients with and without DF. In the DF group, a strong positive correlation was found between PAB and NLR values (r = 0.449; *p* < 0.05). In contrast, no significant correlations were found between NLR and OS biomarkers in patients without DF (data not shown). 

A binary logistic regression analysis revealed that increased levels of both PAB and NLR were significantly associated with the presence of DF (Table 3), while decreased activity of SOD was not (OR = 0.964; 95%CI: 0.930–1.000; *p* = 0.05). The observed associations of PAB and NLR remained significant even after adjustment for age, smoking status, and HbA_1c_ levels. After adjustment for diabetes duration, PAB remained a significant predictor of DF, while the association between the NLR ratio and DF was lost (*p* = 0.068). In the multivariable model that included all confounding variables, PAB remained significantly associated with DF (OR = 1.072; 95%CI: 1.008–1.140; *p* < 0.05) while NLR did not (*p* = 0.066).

**Figure 2:**
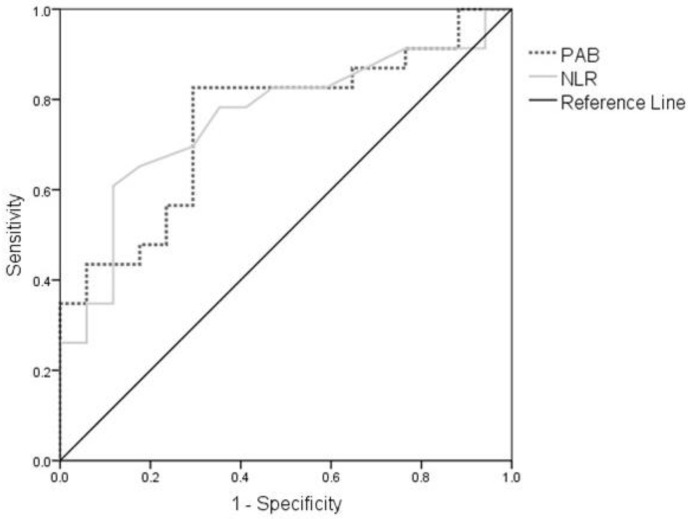
ROC curve for PAB and NLR. Abbreviations: ROC, Receiver operating characteristic curve; PAB, prooxidant-antioxidant balance; NLR, neutrophils-to-lymphocyte ratio.

Finally, we examined the diagnostic accuracy of PAB and NLR to identify the patients with DF. The diagnostic accuracy of both PAB (AUC = 0.741; 95%CI: 0.590–0.893; *p* < 0.01) and NLR (AUC = 0.760; 95%CI: 0.608–0.911; *p* < 0.01) was acceptable. ROC curves for PAB and NLR were presented in Figure 2. For the PAB cut-off value of 149 U/L, the sensitivity of the test was 82.6%, while the specificity was 68%. For the NLR cut-off value of 1.9, the sensitivity of the test was 78.3% and the specificity was 64.7%. Furthermore, we found that a combination of PAB and NLR additionally improved the diagnostic accuracy for DF (AUC = 0.790; 95% CI: 0.651–0.930; *p* < 0.01).

## 4. Discussion

In this study, we found that patients with DF are characterized by proinflammatory and prooxidative states, which are reflected by elevated levels of inflammatory biomarker NLR, increased serum prooxidants, and a concomitant decrease of antioxidative mechanisms. In addition, our data showed that elevated PAB and NLR are associated with the presence of DF. We wish to clarify that in the present study we included all patients consecutively diagnosed with DF in a 4-month period in the General Hospital. Given that the study was conducted during the COVID-19 pandemic, we only included patients who did not have a history of COVID-19 in order to avoid the impact of SARS-CoV-2 virus infection on inflammation or oxidative stress, as well as the impact of COVID-19 itself on the course of diabetes. As a result of this, our study has a relatively small sample size of 23 patients with DF, although previous studies in DF patients also had similar sample sizes.

Diabetic foot is one of the most serious complications of diabetes, which reduces the quality of life and life expectancy of the patients. For this reason, timely recognition of risk factors in order to improve prevention of DF development and delay amputation is of critical importance. Among the risk factors for DF development, a longer duration of diabetes has been recognized as one of the principal contributors [2]. In line with the previous statement, DF patients included in our study suffered from diabetes longer than patients without DF (Table 1). In both groups, the majority of patients had poorly regulated diabetes and hypertension, a common T2DM comorbidity (Table 1). Similarly, the prevalence of obese patients in the DF group did not differ from the group without DF. This finding is consistent with the study of Lavery et al. [29], which demonstrated that obesity, although a risk factor for T2DM, is not directly associated with the development of foot ulcerations. 

Hyperglycemia due to poor metabolic control is recognized as the main initiator of cellular damage, which precedes the development of DF [10]. In the current study, significantly lower HbA_1c_ levels were observed in the DF group (Table 1), and such an unexpected result was further ascribed to a high percentage of anemic patients in the DF group (Table 2). The most common form of anemia in DF patients is anemia of chronic disease [30]. Namely, chronic inflammation is characterized by cytokine overproduction, which may suppress the synthesis of erythropoietin [31]. In addition, intestinal absorption of iron is inhibited by hepcidin upregulation, which reduces its availability for erythropoeisis [32]. Thus, inability to increase erythropoietin concentration in response to decreased Hgb is considered to be the main cause of anemia in DF [30]. Our data clearly demonstrate the limitation of HbA_1c_ testing in patients with anemia. It should also be mentioned that, according to ADA criteria, the introduction of insulin into the treatment of T2DM begins when—despite the use of two different OAD medications—HbA_1c_ levels remain above target values (<7%). In our study, patients with DF mostly received insulin therapy (Table 1), which may also contribute to lower HbA_1c_ levels in this group. 

Although patients with T2DM are characterized by enhanced OS [7,8,9], the results of the current study showed an even more disturbed redox status in patients with DF, as indicated by increased levels of PAB—with concomitantly reduced activities of SOD (Figure 1). PAB is a biomarker of the balance between serum prooxidants and antioxidants and its elevation indicates ROS overproduction [33]. Some previous studies have suggested that redox imbalances could have a pivotal role in the development and delayed healing of DF ulcers [11]. Accordingly, serum OS biomarkers were recently recognized as potentially valuable tools in diagnostics and management of DF [11,12]. The results of the study by Yarahmadi et al. [13] showed that PAB levels in DF patients highly exceed reference values. The authors also found a positive correlation between PAB levels and hsCRP [13], which indicates that both oxidative stress and inflammatory processes are exacerbated in patients with DF. It is firmly established that hyperglycemia contributes to the development of OS by overproduction of superoxide anion radicals through the mitochondrial electron transport chain [10]. Another potential explanation for increased PAB could be the fact that 40% of our patients with DF were smokers, while the number of smokers was markedly lower in the group without DF (Table 1). Although smoking is a strong risk factor for the development of DF ulcerations and subsequent amputations [4], it had no impact on the observed association between PAB and DF (Table 3), suggesting the role of other potential sources of ROS generation during DF development.

Previous studies have established the depletion of antioxidant protective mechanisms as an important risk factor for the development of diabetic complications. So far, the activity of SOD in patients with DF has been investigated to a lesser extent. Bolajoko et al. [15] reported reduced SOD activity in patients with DF compared to healthy controls. Our current study further demonstrated that patients with DF had lower SOD activity than DM patients without this complication (Figure 1). The finding of reduced SOD activity in patients with DF implies a potential exhaustion of the enzyme activity due to superoxide anion radical overproduction. Indeed, superoxide anion radicals are considered the main contributors to oxidative damage of foot tissue [34]. The observed reduced activity of SOD in patients with DF may also be a consequence of glycation of the enzyme, since it has been demonstrated that the degree of glycation is proportional to the duration of diabetes [31], which was significantly higher in our DF group (Table 1). Another important finding of our study is the reduced serum content of SHG in DF patients who underwent amputation. Gluthatione is one of the main contributors to the serum pool of thiols, which is usually estimated as a content of total SHG. Reduced gluthatione is among the most important endogenous antioxidants due to its scavenger role in neutralizing electrophilic and oxidant species. Other authors also reported depleted levels of reduced gluthatione in patients with DF [14]. Our data suggest that the activity of antioxidant mechanisms further decrease with DF progression toward amputation, which should be confirmed in further studies. 

In recent years, the importance of low-grade inflammation in the development of DF has been increasingly recognized [16]. The NLR index has been previously demonstrated as a potentially useful biomarker of systemic inflammation, but also as a predictor of future development of CVD [19]. More recently, its significance has been reported in diabetes [20]. The NLR index comprises the main components of the chronic inflammatory response. In this respect, an increased neutrophil proportion indicates an active inflammatory process, while a reduction of the lymphocyte proportion reflects an inadequate immune response, which is one of the characteristics of T2DM [35]. Recent studies have shown that NLR values are higher in patients with peripheral neuropathy and foot ulcerations compared to patients without DF [20,21,35]. Consistent with these findings are the results of our study, which showed that the NLR index was significantly higher in patients with DF compared to those without this complication (Table 2). Taken together, these results suggest a further increase of NLR values with the exacerbation of the disease. In a recent follow-up study of patients with DF ulcers, a marked difference in NLR between the patients with complete healing ulcers and those who underwent amputation was demonstrated. On the other hand, the patients with chronic wounds and those who underwent major amputation had similar NLR [22]. These data suggest that NLR determination might be useful in the prediction of DF outcome. In our current study, there is no difference in NLR between the DF patients who did and did not undergo amputation, which could be the consequence of its cross-sectional design, as well as due to a relatively small number of included patients. Future studies with a larger sample size would be needed to assess whether NLR may be used as a biomarker of amputation risk. 

Furthermore, we found a positive correlation between PAB and NLR, which may reflect the interactive role of OS and inflammation in the development of DF. Namely, enhanced OS triggers the activation of the nuclear factor kappa B (NF-κB) pathway, which leads to increased production of proinflammatory mediators [16]. On the other hand, infiltration of leukocytes to the site of injury further contributes to ROS generation. Among them, stimulated neutrophils are considered to be a major source of ROS as a consequence of the upregulated activities of myeloperoxidase (MPO) and NADPH-oxidase [36]. Thus, elevated NLR might be regarded as an indicator of potential feedback loops between inflammation and OS processes.

Oxidative stress and inflammation affect the wound healing process, but whether these processes could differentiate between an infectious and non-infectious state is still insufficiently explored. Vatankhah et al. [22] recently evaluated the association between NLR and the treatment outcomes in patients with DF and found that higher NLR was associated with higher odds for non-healing ulcers. Since the observed association was independent of main risk factors, including wound infection, the authors suggested that NLR could serve as a biomarker of inflammatory mechanisms per se, rather than infection [22]. Indeed, increased oxidative stress and inflammation are common features of both infectious and non-infectious diseases, such as cardiometabolic diseases, including diabetes mellitus [37,38,39]. 

The results of our study extended previous findings by demonstrating that both PAB and NLR levels are independently associated with DF (Table 3), suggesting a possible additive effect on the risk for DF development. Indeed, both biomarkers had acceptable diagnostic accuracy (Figure 2), which could be further improved by their simultaneous determination. Recently, Chen el al. [40] reported higher NLR values in patients with diabetic neuropathy in relation to patients without this complication. However, they found that NLR had a weak diagnostic accuracy for diabetic neuropathy only in type-1 diabetes mellitus patients [40]. To the best of our knowledge, no previous study investigated the diagnostic accuracy of NLR for DF in patients with T2DM. These data emphasize the need for further studies on the clinical importance of NLR determination in the assessment of diabetic complications in both type-1 and type-2 diabetes mellitus patients.

This study has several limitations. Its cross-sectional design allowed us to demonstrate associations, but conclusions regarding causality cannot be drawn. Due to a lack of wound tissue cultures, we could not determine the impact of infection on the NLR ratio in patients with DF, and consequently whether NLR could differentiate between an infectious and non-infectious state. Further, the treatment of DF and indication for amputation was at the discretion of the experienced treating surgeon and we are unable to provide full data since, for the purpose of the current study, data on the detailed indications for amputation were not collected. We can only assume that the indication for amputation was the extended infection and gangrene, which follows the national guidelines; still, we wish to be fully transparent by stating that the data availability on the indications for surgery would have been valuable to further support our obtained results. In addition, we haven’t collected any data about infection, with the only exception being the information on SARS-CoV-2 infection that was applied as exclusion criteria—since our main aim was to explore the significance of OS and inflammatory biomarkers in patients with DF. It should be noted that NLR is a biomarker of an infection, although in the present study it was evaluated as a biomarker of systemic inflammation. Finally, due to a relatively small sample size, conclusions from the multivariate analysis could be biased, as a larger number of predictors were included in the model.

Still, one of the strengths of this observational study is the fact that it was conducted during the COVID-19 pandemic. We only included patients who did not have a history of COVID-19 in order to avoid the impact of SARS-CoV-2 virus infection on inflammation or oxidative stress, as well as the impact of COVID-19 itself on the course of diabetes, since it has been shown that the consequences of post-COVID syndrome (i.e., “long COVID”) are even more pronounced in patients with diabetes, including persistent tachycardia, sarcopenia, and microvascular dysfunction [41]. 

## 5. Conclusions

In conclusion, the results of our study showed increased PAB levels and NLR in patients with DF. Both PAB and NLR were independently associated with DF and could be suggested in clinical practice to improve the early detection of DF. Further investigations are required to confirm our preliminary findings.

## Figures and Tables

**Table 1:** Demographic and clinical data of study participants.

Parameter	Without DF	With DF	*p*
N	19	23	
Age, years	59.2 ± 12.9	66.74 ± 9.4	<0.05
Gender, male/female, %	57.9/42.1	82.6/17.4	0.098
BMI, kg/m^2^	31.7 ± 5.7	28.7 ± 4.7	0.116
Diabetes duration, years	7.1 ± 5.6	13.7 ± 7.8	<0.05
Obesity, %	69.2	45.5	0.293
Hypertension, %	88.9	77.3	0.427
Smoking, %	18.8	40.9	0.178
HbA_1c_, %	9.6 ± 1.7	7.8 ± 1.3	<0.05
Glucose, mmol/L	10.1 ± 3.7	9.7 ± 2.6	0.665
Therapy			
OAD	43.7	30.4	<0.001
OAD + Insulin	43.8	0	<0.001
Insulin	12.5	69.6	<0.001

Abbreviations: BMI, body mass index; OAD, oral antidiabetic drugs; HbA_1c_, glycated hemoglobin. Data were presented as means ± standard deviations and as relative frequencies. Continuous variables were compared by Student’s *t* test and categorical variables by Chi-square and Fisher Exact tests.

**Table 2:** CBC parameters and NLR index in patients without DF and with DF.

Parameter	Without DF	With DF	*p*
RBC (×10^12^/L)	4.74 ± 0.61	4.04 ± 0.71	<0.05
Hgb, g/L	137.3 ± 19.7	121.2 ± 10.8	<0.05
WBC (×10^9^/L)	7.8 ± 1.8	8.9 ± 2.5	0.123
Neutrophils (×10^9^/L) ^#^	4.0 (3.3–5.3)	5.6 (4.2–6.6)	<0.05
Lymphocytes (×10^9^/L) ^#^	2.4 (2.1–2.8)	1.9 (1.6–2.3)	<0.05
Monocytes (×10^9^/L) ^#^	0.6 (0.5–0.7)	0.7 (0.5–0.8)	0.331
NLR ^#^	1.6 (1.3–2.1)	2.8 (2.0–4.3)	<0.01

Abbreviations: RBC, red blood cells; Hgb, hemoglobin; WBC, white blood cells; NLR, neutrophils to lymphocytes ratio. Data were presented as means ± standard deviations and compared by Student’s *t* test variables. ^#^ Data were presented as medians (interquartile ranges) and compared by Mann-Whitney U-test.

**Table 3:** Logistic regression analysis for the association of PAB and NLR with DF.

	PAB		NLR	
	OR (95% CI)	*p*	OR (95% CI)	*p*
Univariate	1.026 (1.005–1.048)	<0.05	2.812 (1.206–6.568)	<0.05
Adjusted for:				
Age (years)	1.024 (1.003–1.046)	<0.05	2.684 (1.106–6.513)	<0.05
Diabetes duration (years)	1.043 (1.008–1.080)	<0.05	2.915 (0.924–9.196)	0.068
Smoking	1.035 (1.009–1.062)	<0.05	2.621 (1.101–6.230)	<0.05
HbA_1c_ (%)	1.057 (1.014–1.102)	<0.05	4.632 (1.090–19.677)	<0.05

Abbreviations: OR, odds ratio; CI, confidence interval.

## Data Availability

The data presented in this study are available on request from the corresponding author. The data are not publicly available due to ethical regulations.
